# Anion Binding Studies on Receptors Derived from the Indolo[2,3-*a*]carbazole Scaffold Having Different Binding Cavity Sizes

**DOI:** 10.3390/s140814038

**Published:** 2014-07-31

**Authors:** Guzmán Sánchez, David Curiel, Alberto Tárraga, Pedro Molina

**Affiliations:** Departmento de Quimica Orgánica, Facultad de Química, Universidad de Murcia. Campus de Espinardo, 30100 Murcia, Spain; E-Mails: snxz@um.es (G.S.); atarraga@um.es (A.T.)

**Keywords:** supramolecular chemistry, anions, indolocarbazole ring, benzoate anion, fluorescence spectroscopy, absorption spectroscopy

## Abstract

The indolo[2,3-*a*]carbazole scaffold is a fused polyheteroaromatic system bearing two NH groups which suitably converge as hydrogen bond donor sites for the recognition of anions. A simple derivatisation of the indolocarbazole system at positions 1 and 10 with different functional groups, namely alcohols and amides, has contributed to modulate the anion binding selectivity and sensibility. A particularly good response has been obtained for the benzoate anion.

## 1. Introduction

Anion-mediated processes are ubiquitous in Nature. For instance, it has been proved that misregulation of certain anion levels is associated with physiological malfunctions [1–3]. Furthermore, the polluting effect of anions also represents a matter of concern [4–6]. Consequently, the topics of anion complexation, sensing and/or transport have gained much relevance within the area of supramolecular chemistry [7–11].

In this regard the role of benzoate anion is particularly interesting since it has been widely employed as a conservative in food, toothpastes or medicinal syrups due to its antimicrobial properties, low toxicity and flavor [12,13]. Besides, benzoates are commonly used as yeast and mould inhibitors and also against a wide number of bacteria.

Because of the importance of this anion, its detection has become an essential issue. Despite the availability of certain analytical methods based on chromatography [14,15] or capillary electrophoresis [16], it would be desirable to obtain synthetic receptors which could be applied to the sensing of benzoate by simple molecular recognition processes.

**Figure 1.**
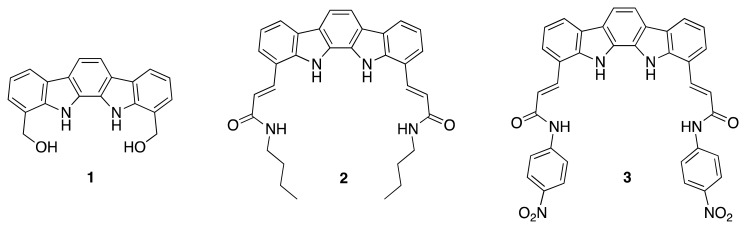
Structures of indolocarbazole-based receptors.

Due to our interest in the design of pyrrole-based receptors with a highly preorganised structure [17–19], we focused our attention in the indolo[2,3-*a*]carbazole system. This fused pentaheterocyclic ring system presents two well-oriented NH groups which define an arch-shaped cavity with a good geometrical match with the “Y”-shaped carboxylate anions. In this regard, although very interesting results have been have been reported concerning the use of indolo[2,3-*a*]carbazole as an anion receptor [20–26], it is quite surprising that not many efforts have been put into the derivatisation of this promising scaffold. In this context, we present herein the synthesis of a series of indolo[2,3-*a*]carbazole receptors with different functional groups and cavity sizes (Figure 1) and their evaluation as anion sensors.

## 2. Results and Discussion

### 2.1. Synthesis of Receptors **1–3**

**Scheme 1.**
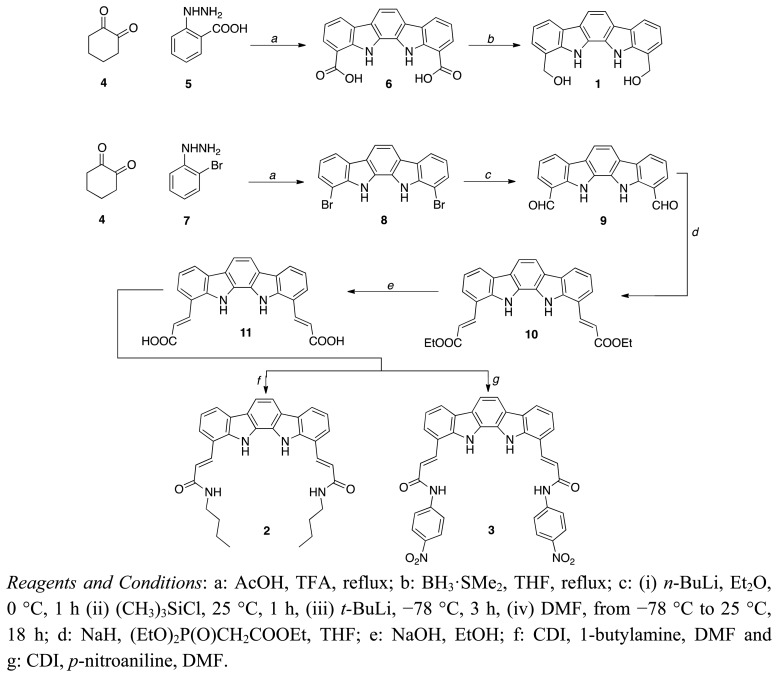
Synthesis of the receptors.

The synthesis of the studied receptors was carried out as depicted in Scheme 1. The access to the indolo[2,3-*a*]carbazole scaffold was accomplished using a one-pot procedure involving a double Fischer indolisation between **4** and the corresponding phenylhydrazine in AcOH/TFA mixtures. The reaction with *o*-hydrazinobenzoic acid, **5**, led to the formation of the dicarboxylic acid **6**, which was treated with BH_3_·SMe_2_ in THF to obtain the diol **1.**

Using a similar synthetic route, the reaction of 1,2-cyclohexanedione (**4**) with *o*-bromo-phenylhydrazine enabled the isolation of the *bis*-brominated derivative **8**. The protection of the indolocarbazole NH groups with TMSCl, followed by the lithiation of the heteroaromatic system and subsequent reaction with DMF produced the dialdehyde **9**. The expansion of the binding cavity was achieved *via* a Horner-Wadsworth-Emmons reaction on **9**, which stereospecifically led to the α,β-unsaturated diester **10**. Finally, hydrolysis of ester groups led to the dicarboxylic acid **11** and reaction of the latter with butylamine or *p*-nitroaniline in the presence of 1,1′-carbonyldiimidazole (CDI) in DMF produced receptors **2** and **3**, respectively.

### 2.2. Binding Studies

**Figure 2.**
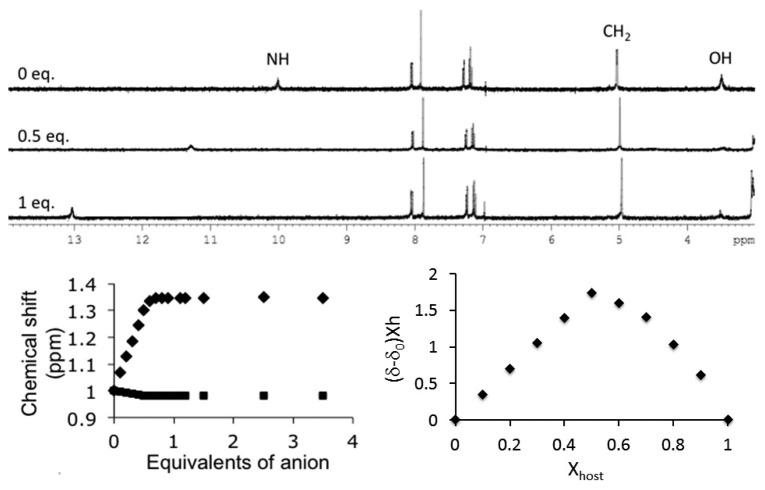
Evolution of ^1^H-NMR spectra upon titration of **1** with acetate anions in CD_3_CN ([**1**] = 2 × 10^−3^ M). Inset: Titration isotherms and Job plot (♦, NH; ▪, CH_2_).

Initially, binding studies on receptor **1** were performed by ^1^H-NMR in CD_3_CN (Figure 2). When 1,10-di(hydroxymethyl)-indolo[2,3-*a*]carbazole, **1**, was titrated with a series of anions, namely acetate, benzoate, dihydrogenphosphate, hydrogenpyrophosphate, chloride and bromide, the expected downfield shift was detected for the NH protons which formed hydrogen bonds with the anionic guests.

Unfortunately, the OH protons rapidly exchanged and disappeared after the addition of the first aliquots of anions. Nevertheless, the peak ascribed to the CH_2_ protons still exhibited a subtle shift induced by the complexed anion. The detected evolution of the NMR peaks clearly evidenced a preferential binding towards oxyanions.

**Figure 3.**
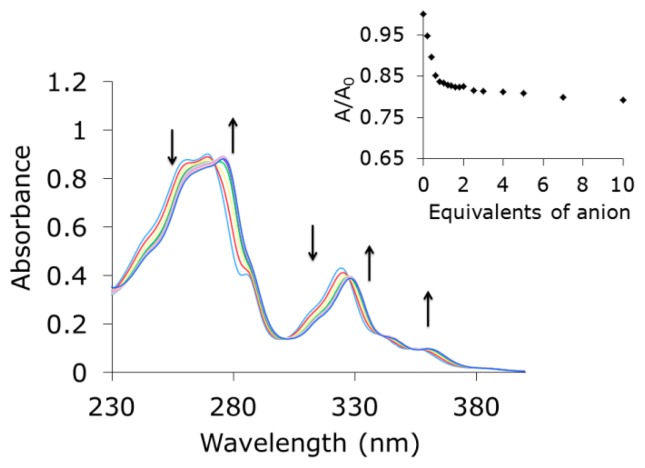
Evolution of absorption spectra upon addition of acetate in acetonitrile. Inset: titration isotherm at λ = 324 nm. [**1**] = 2 × 10^−5^ M.

The fused polyheteroaromatic structure of indolocarbazole allows that it can be simultaneously used as binding unit and as signaling unit. The UV-vis spectrum of **1** showed five bands at λ = 261 nm (ε = 43,000 cm^−1^·M^−1^), λ = 269 nm (ε = 45,000 cm^−1^·M^−1^), λ = 285 nm (ε = 19,500 cm^−1^·M^−1^), λ = 324 nm (ε = 21,500 cm^−1^·M^−1^), and λ = 359 nm (ε = 4300 cm^−1^·M^−1^) assigned to the π-π* transitions of the indolocarbazole system (Figure 3). Accordingly, titration experiments carried out by absorption spectroscopy showed a bathochromic shift of all the bands in the spectrum upon anion complexation. Additionally, four isosbestic points could be detected indicating the establishment of a well-defined equilibrium between host and guest [27].

Job plot analysis of the titrated anions confirmed a 1:1 stoichiometry for most of them (see Supplementary Information). However, a different evolution was detected for the hydrogen-pyrophosphate anion. This caused the saturation of the binding curve right after the addition of 0.5 equivalents, which could be related to the formation of a 2:1 (H:G) complex due to the larger size of this anion. This result was further confirmed by the Job plot, which displayed a minimum at a value of 0.66 for the molar fraction of the receptor. The previously mentioned preference towards oxyanions was corroborated by the binding constants determined from non-linear regression of the experimental binding curves. In this regard, the angular and tetrahedral geometries of acetate, benzoate, dihydrogenphosphate and hydrogenpyrophosphate anions enabled a better geometrical correspondence with the di(hydroxymethyl)indolocarbazole **1**.

**Figure 4.**
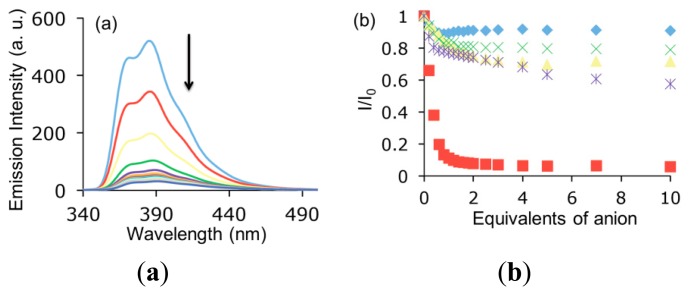
(**a**) Evolution of emission spectra upon addition of benzoate in acetonitrile, [**1**] = 2 × 10^−5^ M. (**b**) Titration isotherms: 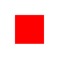 benzoate, 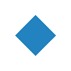 acetate, 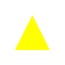 fluoride, 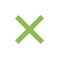 dihydrogenphosphate and 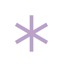 hydrogenpyrophosphate.

The weak sensitivity resulting from the UV-vis experiments, led us to the study of anion complexation by the more sensitive fluorescence spectroscopy (Figure 4). Concerning the emission spectrum (λ_exc_ = 300 nm) of diol **1**, it showed a band at 387 nm accompanied by a shoulder at 371 nm, ascribed to the π-π* transitions of the indolocarbazole system. The titration experiments with AcO^−^ and H_2_PO_4_^−^ only caused a subtle decrease in the fluorescence spectrum along with a weak bathochromic shift. As far as the HP_2_O_7_^3−^ anion is concerned, a two-phase sigmoidal profile proved the higher 2:1 stoichiometry discussed above. Conversely, a more sensitive response was obtained for the benzoate anion which provoked the almost complete quenching of the indolocarbazole emission. Finally, a comparison of the diol **1** with the plain indolo[2,3-*a*]carbazole evidenced an increase in the stability of the complexes as a result of the attached hydroxymethyl groups (Table 1).

Motivated by the promising results obtained from a very simple functionalisation of the indolocarbazole skeleton, we decided to examine the possibility of expanding the size of the indolocarbazole binding cavity. Since most of the reported 1,10-disustituted indolocarbazole-based anion receptors describe a conformationally restricted cavity, the widening of the receptor represents an unexplored option. As it has been previously anticipated our approach consisted in the synthesis of *(E)*-double bonds appended to the polyheteroaromatic system. Subsequently amide functional groups were incorporated to the expanded π-conjugated system.

**Figure 5.**
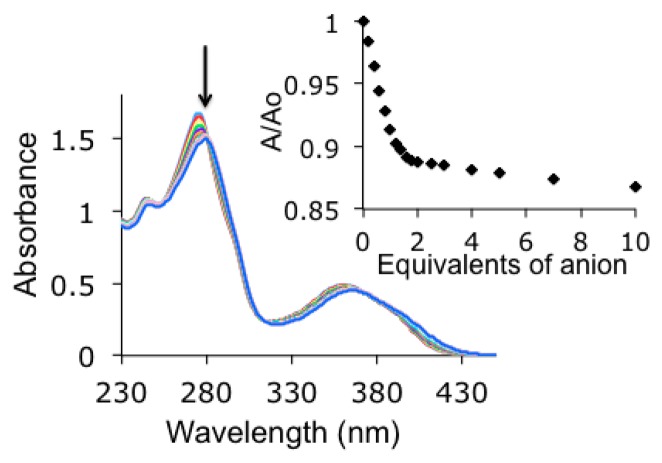
Evolution of absorption spectra of **2** upon addition of acetate in acetonitrile. Inset: titration isotherm at λ = 360 nm. [**2**] = 2 × 10^−5^ M.

Preliminary studies with the *N*-butyl amide **2**, denoted a red shift of the UV-vis spectrum as a result of the extended conjugation. Once again, the titration with the series of tested anions (AcO^−^, BzO^−^, H_2_PO_4_^−^, Cl^−^ and Br^−^) produced a weak bathochromic shift (Figure 5). It is worth highlighting that differently from the diol **1**, the diamide **2** could bind halide anions such as chloride and bromide. Regarding the experiment with the HP_2_O_7_^3−^ anion, it described a complex titration isotherm denoting a dissimilar binding mode than the rest of the anions. Anyhow, the association constants determined for compound **2** manifested a lack of selectivity for any of the assayed anions (Table 1).

**Figure 6.**
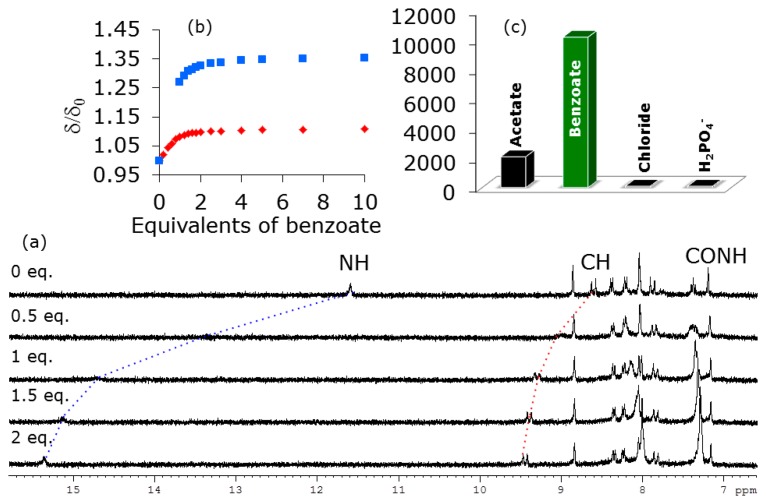
(**a**) Evolution of ^1^H-NMR spectra of 3 upon addition of benzoate in DMSO-*d_6_*. (**b**) Titration isotherm for pyrrolic NH ( 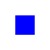) and olefinic CH ( 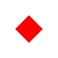). [3] = 2 × 10^−3^ M. (**c**) Calculated binding constants.

With the aim of investigating the effect of the environment on the anion binding ability of the expanded indolocarbazole receptors, the diamide **3** was analysed in a more competitive solvent such as DMSO. The ^1^H-NMR titrations confirmed the expected downfield shift of the pyrrolic NHs taking part in the complexation of the anions (Figure 6). Although these peaks vanished during the initial part of the titration, they came up again as the titration progressed. Interestingly, the olefinic protons in receptor **3** also showed a downfield shift caused by the deshielding effect of hydrogen bonding the anionic guests. Nevertheless, the peaks ascribed to the amide NHs did not display any significant displacement, which could be interpreted in terms of the perhaps too long distance from the center of the binding cavity. In any case, it was the benzoate anion which formed more stable complexes compared to the rest of the series of anions. Therefore, the increase in the polarity of the environment produced a noticeable improvement in the binding selectivity which was evidenced even for the two analysed carboxylate anions, *i.e.*, benzoate (K_assoc._ = 1.02 × 10^4^ M^−1^) and acetate (K_assoc._ = 2.09 × 10^3^ M^−1^).

**Figure 7.**
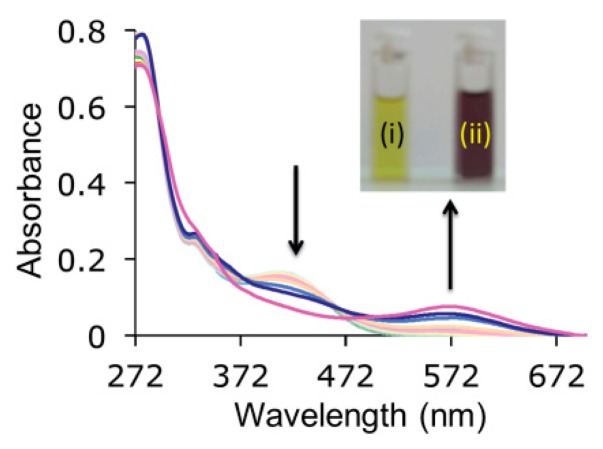
Evolution of UV-vis spectra of **3** (10^−5^ M) upon titration with hydrogenpyrophosphate in DMSO. Inset: Colour change of receptor **3** (i) after the addition of HP_2_O_7_^3−^ (ii).

Concerning the UV-vis experiments, the higher competitiveness of the environment, joined to the more diluted concentration of the samples, resulted in a negligible response for most of the studied anions. Nevertheless, those anions with a stronger basicity induced a colour change in the solution which, in the case of hydrogenpyrophosphate, was especially meaningful since the solution turned from yellow to dark red (Figure 7). Control experiments performed with a strong base such as TBAOH, confirmed that the detected colour change corresponded to a deprotonation process. Additionally, the presence of the *p*-nitrophenyl groups in **3**, favoured the colorimetric response which had not been observed for other indolocarbazole receptors.

By virtue of very simple structural modifications in the binding cavity of the indolocarbazole a modulation of the selectivity and sensitivity of a multichannel response has been achieved.

## 3. Experimental Section

### 3.1. General Information

Solvents were dried following the usual protocols. THF, Et_2_O and toluene were distilled from sodium wire with benzophenone indicator; CH_3_CN and CH_2_Cl_2_ were distilled from CaCl_2_; EtOH and MeOH were distilled from magnesium and stored with molecular sieves. All anions were employed as their tetrabutylammonium salts. Unless stated otherwise, all reactions were carried out under nitrogen atmosphere. Column chromatography was run with silica gel 60 Å CC 70–200 μm as stationary phase and using HPLC grade solvents. Melting points were measured in a Reichert instrument and are not corrected. ^1^H-NMR, ^13^C-NMR and NOESY experiments were recorded on a Bruker AV200, AV300, AV400 or AV600 instruments. Chemical shifts are referred to the residual peak of the solvent. In the experimental data “bp” stands for broad peak and “Cq” for quaternary carbon atom. Mass spectrometry was recorded on HPLC-MS TOF 6220 instrument. Absorption spectra were recorded on a Cary 5000 UV-vis-NIR spectrophotometer. Emission spectra were recorded on a Cary Eclipse spectrophotometer. Microanalyses were performed on a Carlo Erba 1108 instrument. All binding constants were calculated by a non-linear fitting procedure using the software SPECFIT/32 Global Analysis System.

### 3.2. Synthesis

#### Indolo[2,3-a]carbazole-1,10-dicarboxylic acid (**6**)

1,2-Cyclohexanedione (1.0 g, 8.90 mmol) and *o*-hydrazinobenzoic acid (5.3 g, 26.70 mmol) were stirred in acetic acid (150 mL) at room temperature for 6 h. Then, trifluoroacetic acid (10 mL) was added and the suspension was refluxed overnight. After this time, the reaction was filtered while hot and the resulting yellow solid washed with acetic acid (2 × 25 mL), water (2 × 50 mL) and dried *in vacuo* yielding the desired product as a light yellow solid (0.6 g, 20%). Mp: >300 °C ^1^H-NMR (200 MHz, DMSO-*d_6_*); δ (ppm): 7.30 (t, 2H, *J* = 7.6 Hz); 8.00–8.37 (m, 4H); 8.47 (d, 2H, *J* = 7); 12.22 (s, 2H); 13.18 (br, s, 2H). ^13^C-NMR (50 MHz, DMSO-*d_6_*): δ (ppm): 112.3 (CH); 113.1 (CH); 118.4 (CH); 120.1 (CH); 125.0 (Cq); 125.2 (Cq); 126.0 (Cq); 126.9 (Cq); 138.3 (Cq); 168.0 (C=O). MS *m/z (%):* 344 (M^+^, 70), 326 (M^+^-H_2_O, 94), 308 (M^+^-2H_2_O, 77). Anal. Calc. for C_20_H_12_N_2_O_4_: C, 69.76; H, 3.51; N, 8.14. Found: C, 69.51; H, 3.81; N, 8.42.

#### 1,10-Bis-(hydroxymethyl)-indolo[2,3-a]carbazole (**1**)

Indolo[2,3-*a*]carbazole-1,10-dicarboxylic acid (**6**, 430 mg, 1.25 mmol) was disolved in dry THF (80 mL) under a nitrogen atmosphere and the mixture was heated at reflux temperature. Then, BH_3_·SMe_2_ 10M in THF (0.75 mL, 7.5 mmol) was added and the reaction was refluxed overnight. After such time, the mixture was cooled down to 0 °C using an ice bath and HCl 4N was carefully added (20 mL). Then, the solvent was evaporated in a rotary evaporator and the crude was extracted with EtOAc (3 × 30 mL), dried over anhydrous Na_2_SO_4_, filtered off and evaporated to obtain a yellow solid. After rinsing with ether (3 × 15 mL), the expected product was isolated as a yellow solid (220 mg, 56%). Mp: >300 °C ^1^H-NMR (400 MHz, DMSO-*d_6_*); δ (ppm): 4.95 (s, 4H); 5.45 (t, 2H, *J* = 5.2 Hz); 7.15 (t, 2H, *J* = 7.2 Hz); 7.30 (d, 2H, *J* = 6.4 Hz); 7.88 (s, 2H); 8.00 (d, 2H, *J* = 7.6 Hz); 11.24 (s, 2H). ^13^C-NMR (100 MHz, DMSO-*d_6_*); δ (ppm): 61.5 (CH_2_); 111.5 (CH); 118.5 (CH); 118. 7 (CH); 119.8 (CH); 122.7 (CH); 123. 8 (Cq); 125.0 (Cq); 125.6 (Cq); 137.0 (Cq). MS (EI) *m/z (%):* 317 (M^+^ + 1, 7), 316 (M^+^, 35). Anal. Calc. for C_20_H_16_N_2_O_2_: C, 75.93; H, 5.10; N, 8.86. Found: C, 76,24; H, 4.77; N, 8.69

#### 1,10-Dibromoindolo[2,3-a]carbazole (**8**)

A slurry of cyclohexanedione (1000 mg, 8.9 mmol) and 2-bromophenylhydrazine (5970 mg, 26.9 mmol) in glacial AcOH (150 mL) was stirred at room temperature for 6 h. Once the reactants had dissolved, trifluoroacetic acid (10 mL) was added and the mixture was refluxed overnight. After this time the reaction was filtered while hot and the filtrates were poured into ice. The resulting precipitate was filtered, dried *in vacuo* and chromatographed with a polarity gradient from hexane/THF (9/1) to hexane/THF (6/1). The desired compound was isolated as a pale yellow solid (1150 mg, 32%). Mp: 281–283 °C. ^1^H-NMR (400 MHz, DMSO-*d_6_*); δ (ppm): 7.18 (t, 2H, *J* = 7.8 Hz); 7.64 (d, 2H, *J* = 7.8 Hz); 7.98 (s, 2H); 8.20 (d, 2H, *J* = 7.8 Hz); 11.30 (s, 2H). ^13^C-NMR (100 MHz, DMSO-*d_6_*); δ (ppm): 103.8 (CH); 112.84 (CH); 119.4 (CH); 120.5 (CH); 120.6 (Cq); 125.1 (Cq); 125.4 (Cq); 126.9 (Cq); 137.1 (Cq). MS (EI) *m/z (%)*:412 (M^+^, 37), 414 (M^+^+2, 100), 416 (M^+^+4, 33). Anal. Calc. for C_18_H_10_Br_2_N_2_: C, 52.21; H, 2.43; N, 6.76. Found: C, 52.55; H, 2.50; N, 6.51.

#### 1,10-Diformylindolo[2,3-a]carbazole (**9**)

1,10-Dibromoindolo[2,3-*a*]carbazole (**8**, 600 mg, 1.45 mmol) was disolved in dry ether under nitrogen atmosphere and cooled to 0 °C in an ice bath. Then, butyllithium (2.5 M, 2.50 mL, 3.48 mmol) was incorporated into the mixture which was stirred under these conditions for 1h. Next, trimethylchlorosilane (0.45 mL, 3.48 mmol) was added and the reaction was stirred for one more hour at room temperature. After that, the mixture was cooled down to −78 °C and *tert*-butyllithium (1.6 M, 4.14 mL, 3.48 mmol) was carefully added. The reaction was then stirred for 3 h and the temperature was slowly increased to 0 °C during this time. Then, the temperature was lowered again to −78 °C and anhydrous DMF (0.8 mL, 10.34 mmol) was added. Finally, the mixture was stirred overnight while the temperature slowly reached room temperature. After that time, the reaction was cooled to 0 °C and HCl (4N, 30 mL) was added dropwise. The mixture was stirred for 40 min and a yellow precipitate appeared. That solid was filtered off and washed with water (50 mL). The desired product was then isolated as a yellow solid (250 mg, 70%). Mp: >300 °C. ^1^H-NMR (400 MHz, DMSO-*d_6_*); δ (ppm): 7.44 (t, 2H, *J* = 7.6 Hz); 8.04 (d, 2H, *J* = 7.6 Hz); 8.10 (s, 2H); 8.58 (d, 2H, *J* = 7.6 Hz); 10.26 (s, 2H); 12.58 (s, 2H). ^13^C-NMR (100 MHz, DMSO-*d_6_*); δ (ppm): 112.9 (CH); 118.9 (CH); 119.9 (CH); 120.3 (CH); 125.1 (Cq); 126.4 (Cq); 126.8 (Cq); 131.6 (Cq); 135.8 (Cq); 193.5 (C=O). MS (EI) *m/z (%):* 313 (M^+^ + 1, 80). Anal. Calc. for C_20_H_12_N_2_O_2_: C, 76.91; H, 3.87; N, 8.97. Found: C: 76.69; H, 4.03; N, 8.65.

#### 1,10-Bis-(2-ethoxycarbonyl-(E)-vinyl)indolo[2,3-a]carbazole (**10**)

Sodium hydride (78 mg, 3.25 mmol) was suspended in dry THF (25 mL) under nitrogen atmosphere at 0 °C. Triethylphosphonoacetate (0.56 mL, 2.85 mmol) disolved in dry THF was then added dropwise and the reaction was stirred for 30 min. After that time, a solution of 1,10-diformylindolo[2,3-*a*]carbazole (**9**, 400 mg, 1.3 mol) in dry THF (20 mL) was added dropwise and the mixture was stirred for 16 h. Then, the reaction was quenched with water (30 mL), THF was evaporated and the aqueous layer was extracted with EtOAc (3 × 20 mL). After the aqueous workup, the resulting crude was further chromatographed with EtOAc and the desired product was isolated as a yellow solid (500 mg, 93%). ^1^H-NMR (200 MHz, DMSO-*d_6_*); δ (ppm): 1.34 (t, 6H, *J* = 7 Hz); 4.30 (q, 4H, *J* = 7 Hz); 6.86 (d, 2H, *J* = 16 Hz); 7.29 (t, 2H, *J* = 7.6 Hz); 7.84 (d, 2H, *J* = 7 Hz); 8.00 (s, 2H); 8.19 (d, 2H, *J* = 16.2 Hz); 8.29 (d, 2H, *J* = 7.8 Hz); 11,37 (s, 2H). ^13^C-NMR (50 MHz, DMSO-*d_6_*); δ (ppm): 14.4 (CH3); 60.3 (CH2); 112.5 (CH); 117.6 (CH); 117.9 (CH); 119.7 (CH); 120.3 (CH); 122.7 (CH); 124.8 (Cq); 125.0 (Cq); 125.9 (Cq); 137.4 (Cq); 140.4 (Cq); 166. 7 (C=O). MS (EI) *m/z (%):* 452 (M^+^, 9), 451 (M^+^*-1*, 100). mp.: 247–249 °C. Anal. Calc. for C_28_H_24_N_2_O_4_: C, 74.32; H, 5.35; N, 6.19. Found: C, 74.58; H, 5.68; N, 5.86.

#### 1,10-Bis-(2-hydroxycarbonyl-(E)-vinyl)indolo[2,3-a]carbazole (**11**)

1,10-*Bis*-(2-ethoxycarbonyl-*E*-vinyl)indolo[2,3-*a*]carbazole (**10**, 370 mg, 0.9 mmol) was dissolved in EtOH (60 mL) and cooled to 5 °C. Then, NaOH (220 mg, 5.4 mmol) dissolved in water (20 mL) was added and the mixture was refluxed for 10 h. After that time, the mixture was acidified with HCl (4N, 50 mL) to yield the expected product as a yellow precipitate (360 mg, 90%). Mp: >300 °C; ^1^H-NMR (300 MHz, DMSO-*d_6_*); δ (ppm): 6.78 (d, 2H, *J* = 15.9 Hz); 7.29 (t, 2H, *J* = 7.8 Hz); 7.79 (d, 2H, *J* = 7.5 Hz); 8.00 (*s,* 2H); 8.13 (d, 2H, *J* = 16.2 Hz); 8.28 (d, 2H, *J* = 7.8 Hz); 11.365 (s, 2H); 12.18 (pa, *s*, 2H). ^13^C-NMR (75 MHz, DMSO-*d_6_*); δ (ppm): 112.5 (CH); 117.7 (CH); 119.1 (CH); 119.6 (CH); 120.2 (CH); 122.4 (CH); 124.7 (Cq); 124.9 (Cq); 125.8 (Cq); 137.3 (Cq); 140.06 (Cq); 167.9 (C=O). MS (EI) *m/z (%):* 360 (M^+^-2H_2_O, 10%). Anal. Calc. for C_24_H_16_N_2_O_4_: C, 72.72; H, 4.07; N, 7.07. Found: C, 72.91; H, 4.34; N, 7.39.

#### 1,10-Bis-(2-n-butylaminocarbonyl-(E)-vinyl)indolo[2,3-a]carbazole (**2**)

1,10-*Bis*-(2-hydroxy-carbonyl-*E*-vinyl)indolo[2,3-*a*]carbazole (**11**, 110 mg, 0.30 mmol) and CDI (300 mg, 1.85 mmol) were dissolved in dry DMF under nitrogen atmosphere. The mixture was stirred for 6 h at room temperature and freshly distilled *n*-butylamine (0.20 mL, 1.85 mmol) was then added. After stirring overnight and the reaction was quenched with brine (60 mL). A precipitate formed, which was filtered *in vacuo* and washed with water (50 mL). Once dry, the corresponding solid was chromatographed in EtOAc yielding the expected product as a yellow solid (30 mg, 20%). Mp: 283–285 °C. ^1^H-NMR (200 MHz, DMSO-*d_6_*); δ (ppm): 0.79 (m, 6H); 1.37 (m, 8H); 3.25 (m, 4H); 6.64 (bp, *s*, 2H, *J* = 15.2 Hz); 7.17 (t, 2H, *J* = 7.4); 7.47 (d, 2H, *J* = 7.4); 7.87 (s, 2H); 8.20 (m, 4H); 10.31 (s, 2H). ^13^C-NMR (75 MHz, DMSO-*d_6_*); δ (ppm): 13.8 (CH3); 19.8 (CH2); 31.4 (CH2); 38.5 (CH2); 111.8 (CH); 112.6 (CH); 117.9 (CH); 119.7 (CH); 120.3 (Cq); 124.8 (Cq); 126.0 (Cq); 137.6 (Cq); 140.4 (Cq); 166.7 (C=O). MS (EI) *m/z (%):* 506 (M^+^, 2), 433 (98), 362 (37), 152 (100). Anal. Calc. for C_32_H_34_N_4_O_2_: C, 75.86; H, 6.76; N, 11.06. Found: C, 75.59; H, 6.42; N, 10.78.

#### 1,10-Bis-(2-(4nitrophenyl)aminocarbonyl-(E)-vinyl)indolo[2,3-a]carbazole (**3**)

This compound was synthesized using the same procedure as that used for the preparation of **2** with diacid **11** (220 mg, 0.55 mmol), CDI (550 mg, 3.3 mmol) and 4-nitroaniline (500 mg, 3.3 mmol). The compound was isolated as an orange solid (160 mg, 50%). Mp: 283–285 °C. ^1^H-NMR (400 MHz, DMSO-*d_6_*); δ (ppm): 7.19 (s, 2H); 7.37 (t, 2H, *J* = 7.6 Hz); 7.87 (d, 2H, *J* = 15.2 Hz); 8.03–8.04 (m, 4H); 8.20 (d, 2H, *J* = 7.6 Hz); 8.38 (d, 2H, *J* = 7.6 Hz); 8.62 (d, 2H, *J* = 14.8 Hz); 8.85 (s, 2H); 11.70 (bp, 2H). ^13^C-NMR (100 MHz, DMSO-*d_6_*); δ (ppm): 112.7 (CH); 115.7 (CH); 116.9 (CH); 117.4 (Cq); 119.7 (CH); 120.5 (Cq); 123.7 (CH); 124.2 (CH); 125.2 (Cq); 125.9 (Cq); 130.7 (CH); 137.5 (CH); 138.5 (Cq); 143.4 (Cq); 162.3 (C=O). MS (EI) *m/z (%):* 636 (M^+^, 7), 307 (98), 255 (37), 137 (100). Anal. Calc. for C_36_H_24_N_6_O_6_: C, 67.92; H, 3.80; N, 13.20. Found: C, 68.09; H, 3.57; N, 13.56.

## 4. Conclusions

A family of 1,10-disubstituted indolo[2,3-*a*]carbazoles with different hydrogen bond donor groups has been synthesised. Anion binding studies have proved that a straightforward functionalisation of the preorganised indolocarbazole system enabled a noticeable increase in the stability of the complexes. The incorporation of two hydroxymethyl units rendered more sensitive benzoate detection. Additionally, the expansion of the binding cavity through the introduction of two olefins with *E* geometry increased the selectivity towards benzoate anions in DMSO, as evidenced by ^1^H-NMR experiments and offered a selective colorimetric response towards hydrogenpyrophosphate anion.

## Figures and Tables

**Table 1.** Fluorescence association constants for **1** in acetonitrile.^a^

	**AcO^−^**	**BzO^−^**	**F^−^**	**Cl^−^**	**Br^−^**	**H_2_PO_4_^−^**	**HP_2_O_7_^3−^**
Indolocarbazole	1.7 × 10^5^	1.0 × 10^5^	- ^b^	-^c^	- ^c^	4.2 × 10^4^	2.0 × 10^5^
1	8.1 × 10^6^	>10^7^	1.6 × 10^5^	-^c^	- ^c^	8.7 × 10^5^	2.0 × 10^5^ (*K*_11_)
							6.3 × 10^8^ (*β*_21_)
2	4.3 × 10^5^	5.5 × 10^5^	- ^b^	5.6 × 10^5^	4.2 × 10^4^	4.3 × 10^5^	- ^b^
3 ^d^	2.1 × 10^3^	1.0 × 10^4^	- ^e^	-^c^	- ^c^	91	-^e^

a Errors were below 10% in all cases;

b Data could not be accurately fitted;

c Association constant could not be calculated due to too weak binding;

d Association constants determined by ^1^H-NMR in DMSO-*d_6_* [**3**] = 2 × 10^−3^ M;

e Deprotonation process.
